# Dual contraceptive use among women living with HIV on anti-retroviral therapy in Boset district, Ethiopia

**DOI:** 10.3389/fgwh.2025.1510299

**Published:** 2025-04-01

**Authors:** Yohanes Abera Belachwe, Midekso Sento, Melese Negash Gobena, Mihiret Shawel Getahun, Yohannes Mekuria Negussie

**Affiliations:** ^1^ Department of Public Health, Adama General Hospital and Medical College, Adama, Ethiopia; ^2^ Department of Biomedical Sciences, Adama Hospital Medical College, Adama, Ethiopia; ^3^ Department of Nursing, Harambe University, Adama, Ethiopia; ^4^ Department of Nursing, Adama General Hospital and Medical College, Adama, Ethiopia; ^5^ Department of Medicine, Adama General Hospital and Medical College, Adama, Ethiopia

**Keywords:** antiretroviral therapy, dual contraceptives use, women living with HIV, Ethiopia, Boset district

## Abstract

**Background:**

The World Health Organization advocates dual contraceptive methods for women with Human Immunodeficiency Virus (HIV) to prevent unintended pregnancies and sexually transmitted infections (STIs), enhancing education, economic opportunities, and maternal-child health outcomes. However, persistent global challenges stem from inadequate use of dual contraceptives and unsafe sexual practices, resulting in high rates of unintended pregnancies and significant health risks. Hence, this study aimed to assess dual contraceptive use among women living with HIV on anti-retroviral therapy (ART) in Boset District, Ethiopia.

**Method:**

A facility-based cross-sectional study was conducted among 342 women living with HIV from September 12 to October 18, 2023. Participants were selected by systematic random sampling, and data were collected using an interviewer-administered structured questionnaire. The collected data were entered using Epi Info version 7.2.6 and analyzed using SPSS version 26.0. Bivariable and multivariable binary logistic regression analyses were performed to assess the association between the outcome and explanatory variables. In the multivariable analysis, an adjusted odds ratio (AOR) with a 95% confidence interval (CI) was used to show the strength of the association, and the level of significance was set at a *p*-value of <0.05.

**Results:**

The magnitude of dual contraceptive use was 33.7% (95% CI: 28–40). Urban residence (AOR = 2.5; 95% CI: 1.97–8.08), good knowledge about dual contraceptives (AOR = 3.8; 95% CI: 2.36–8.67), STIs history in the past 12 months (AOR = 2.6, 95% CI: 1.25–4.72) and having two or more number of sexual partners, (AOR = 1.9; 95% CI: 1.18–6.68), were factors significantly associated with dual contraceptive use.

**Conclusion:**

One-third of women living with HIV on ART utilized dual contraceptives. Place of residence, knowledge about dual contraceptives, history of STIs in the past 12 months, and number of sexual partners were factors associated with dual contraceptive use. It is essential to implement focused educational programs to increase knowledge about dual contraceptives, alongside expanding access to contraceptive services in rural and underserved areas.

## 1 Introduction

Dual contraceptive utilization involves using a barrier contraceptive to lower the risk of transmitting sexually transmitted infections (STIs) alongside another effective birth control method to prevent unplanned pregnancies (1, 2). The World Health Organization (WHO) advocates for women living with HIV (WLHIV) to employ dual contraceptive methods to prevent unintended pregnancies and STIs (3). Rising rates of STIs are more common in women with HIV than in those without HIV (4). Studies show that unintended pregnancy often happens when individuals engage in sexual intercourse without using contraception, use contraception improperly, or face contraceptive failure despite using it correctly (5).

For women and girls with HIV, using dual contraceptives correctly and consistently leads to better education and economic opportunities, helping to achieve gender equality, empower women, and reduce poverty. It also improves infant and child health by preventing illness and death from unintended pregnancies and STIs, including HIV. This allows for safer, planned pregnancies, benefiting both maternal and child health (6, 7).

Poor utilization of dual contraceptives not only increases the incidence of unplanned pregnancies but also contributes to the spread of HIV/AIDS and other STIs (2, 8). Globally, inadequate use of dual contraceptives and unsafe sexual practices result in over 2 million HIV-infected women becoming pregnant annually, leading to over half a million deaths from pregnancy-related complications (3, 9). Women living with the Human Immunodeficiency Virus often experience unintended pregnancies because women either use ineffective birth control, inadequate education, sexual violence, contraceptive failure, or lack access to family planning services (10). Evidence also suggests that a majority of unintended pregnancies result in unsafe abortion (2).

Studies conducted in different settings indicated that the prevalence of dual contraceptive utilization among WLHIV was 10.7% in Swiss (11), 29.6% in Thailand (3), 27% in Atlanta Georgia (12), 27% in Brazil (13), 32% in Nigeria (14), 33.3% in Cameroon (15), 38.5% in Kenya (16) and 40% in Rwanda (17).

In Ethiopia, the pooled prevalence of dual contraception use among WLHIV was 34.08% (18). The highest proportion of dual contraceptive methods was reported in Bishoftu town which accounts for 56.9% (19). Other studies have found that the prevalence of dual contraceptive utilization among WLHIV was 13.2% in the University of Gondar Hospital (20), 15.7% in Mekelle town (21) 19.4% in Borana district (22), 19.8% in Gebretsadik Shawo Hospital (23), 21.4% in West Shewa Zone (24), 30% in Gimbie town (25).

Previous studies have indicated that factors such as age, marital status, place of residence, educational status, occupational status, having children, fertility desire, counseling on family planning, family support, discussion with a partner, number of sexual partners, disclosure of HIV status, Clusters of differentiation (CD4) cell count were significantly associated with dual contraceptive method utilization (2, 23, 26–28).

Despite several studies on dual contraceptive use among WLHIV in Ethiopia, there remains a limited body of evidence specific to the study setting. Given the regional and contextual differences in healthcare access, contraceptive utilization, and socio-cultural factors, this may limit the direct generalizability of previous findings. Therefore, this study aimed to provide valuable insights by examining the magnitude and factors associated with dual contraceptive use among WLHIV on antiretroviral therapy in Boset District, Ethiopia. The findings will contribute to locally tailored interventions to prevent unintended pregnancies and the transmission of STIs/HIV. Furthermore, the results will bolster planning and decision-making capacities, facilitating collaborative efforts with relevant stakeholders to identify and implement effective solutions to address these issues in the study setting.

## 2 Methods and material

### 2.1 Study design, area, and period

A facility-based cross-sectional study was conducted in the Boset district, central Ethiopia, from September 12 to October 18, 2023. The Boset district is bounded by the Amhara Regional State to the north, Adama Woreda to the west, the Arsi Zone to the south, and Fentale Woreda to the east. The district is comprised of 5 urban and 37 rural kebeles, situated approximately 125 km from Addis Ababa, the capital of Ethiopia. It has a total population of 220,362, of which 112,385 are females (29). The district's healthcare infrastructure includes one hospital, seven health centers, and 30 private clinics. However, only three public health facilities (two health centers and one hospital) provide HIV care and treatment services. A total of 1,610 women were registered as receiving antiretroviral therapy (ART), of whom 764 were women of reproductive age: 206 at Olenchite Hospital, 454 at Olenchite Health Center, and 104 at Bole Health Center.

### 2.2 Study population, and eligibility criteria

All WLHIV who were on ART follow-up in Boset district public health facilities were the source population, while all randomly selected WLHIV who were receiving ART during the study period constituted the study population. The study included women of reproductive age attending ART care services who had attended at least one visit before the study commenced. However, women who were critically ill, unable to communicate, or experiencing mental health issues were excluded. Additionally, women with medical conditions contraindicating the use of contraceptives or other conditions identified based on WHO medical eligibility criteria were excluded to ensure the appropriateness and safety of contraceptive use among participants.

### 2.3 Sample size determination and sampling procedure

A single population proportion formula was used to calculate the sample size with an assumption of 28.8% of the population proportion of dual method of contraceptive use (*p*) from a previous study done in Gonder City (30), 95% confidence level, 5% margin of error and a 10% non-response rate.

$$n=\frac{{\left(z\alpha /2\right)}^{2}p(1-p)}{d^{2}}$$

Where; *n*: Sample size; *Z*: Confidence coefficient; *d*: Margin of error (0.05); *P*: estimation of dual method of contraceptive use rate.

$$n=\frac{0.288(1-0.288){\left(1.96\right)}^{2}}{0.05^{2}}\approx 315$$

After adding 10% contingency for non-response the final sample size of the study becomes 347.

**Figure 1:**
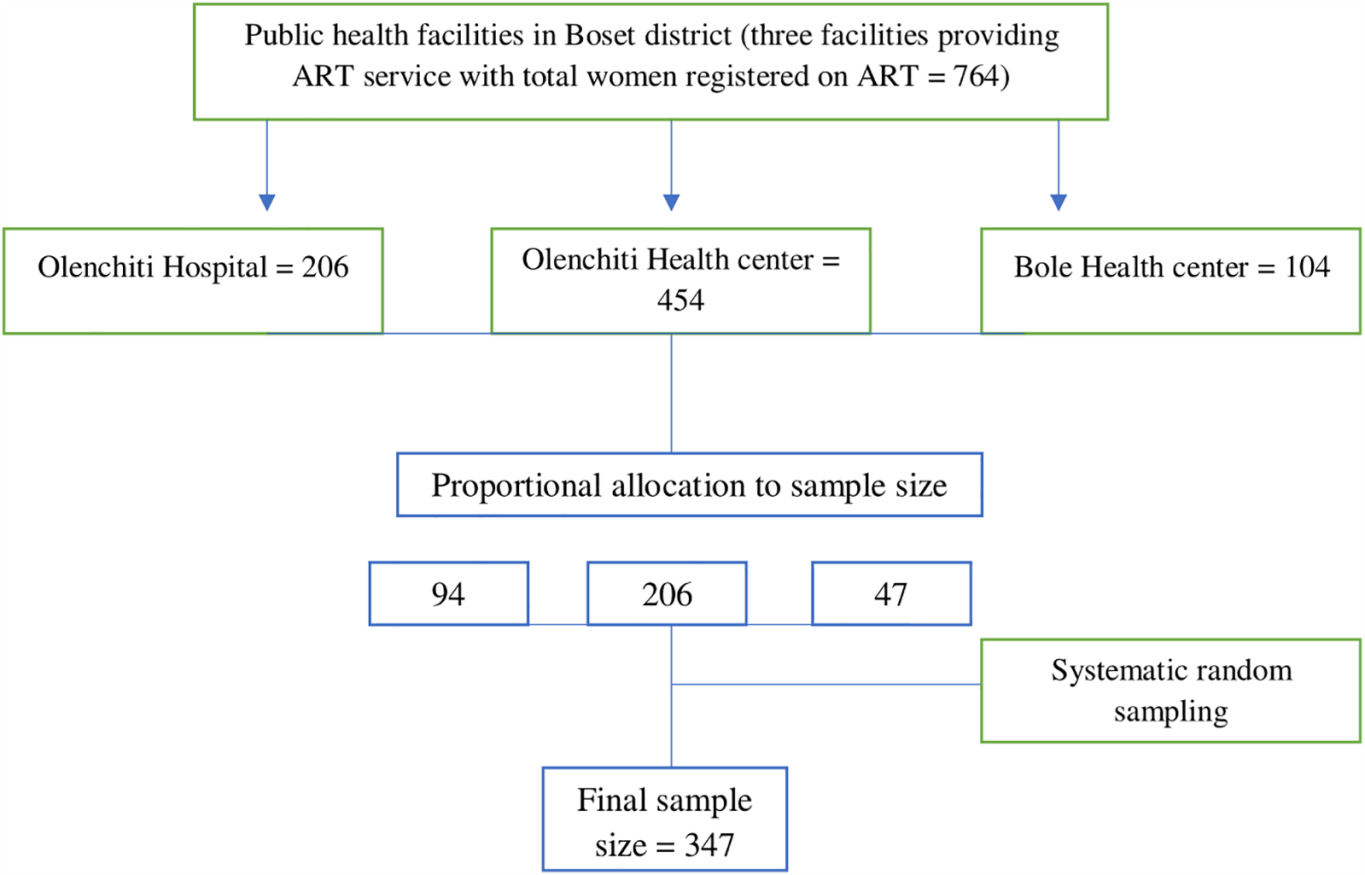
Schematic presentation of sampling procedure to assess dual contraceptive use among women living with HIV on anti-retroviral therapy in Boset District, Ethiopia, 2023.

The study included all three public health facilities providing HIV care and treatment services in the Boset district. The calculated sample size was proportionally allocated to each of these facilities based on the average number of women on ART in the previous three months. Study participants were selected from each chosen health facility using systematic random sampling. The sampling interval at each facility was determined by dividing the average number of women on ART over the previous three months by the required sample size. Thus, the calculated *k*-value becomes two. The index participant was then chosen at random from 1 to 2 in each hospital. Accordingly, every second participant was chosen using a systematic random sampling technique until the required sample size was reached (Figure 1).

Before data collection, eligibility criteria were established to determine the eligibility of WLHIV attending the ART clinic for participation. Once eligible participants were identified, they were assigned sequential numbers based on their arrival order. Participants were then selected at regular intervals (K). To prevent multiple enrollments of the same person, a mark was placed on their medical record.

### 2.4 Study variables

**Dependent variable**

Dual contraceptive use (yes/no).

**Independent variables:**

**Socio-demographic factors:** age, place of residence, religion marital status, ethnicity, Women's educational status, spouse educational status, women occupation. Spouse occupation, and average monthly income.

**Sexual and reproductive health characteristics:** number of children, women's desire to have more children, partner's desire to have more children, ever used any contraceptive method since being HIV-positive, pregnancy since HIV positive, and knowledge about dual contraceptives.

**Clinical and HIV-related characteristics:** STI history in the past 12 months, number of sexual partners, knowing the HIV status of the partner, disclosed HIV status to partner, perceived disclosure of HIV status to a sexual partner is important, Duration on ART, current CD4 cell count, and Current viral load.

### 2.5 Operational definitions

**Dual contraceptive utilization:** The concurrent use of two methods of contraception during sexual encounters in the 12 months preceding the study: a barrier method (male/female condom) and other highly effective modern contraceptive methods (such as the IUCD, contraceptive implants, injections, and pills) (19, 27, 30, 31).

**Knowledge of dual contraceptives:** In this study, the aggregate of eight dichotomized questions assessed knowledge about contraceptive methods. A score of 0 indicated an incorrect answer, while a score of 1 indicated a correct answer. Women who scored at or above the mean were classified as having good knowledge, while those scoring below the mean were classified as having poor knowledge (27).

### 2.6 Data collection procedure and quality control

Data were collected using a pretested, structured, interviewer-administered questionnaire and medical record review. The questionnaires were adapted from various relevant literature with necessary modifications tailored to the specific context of the study (2, 16, 19, 24, 27, 30, 32). A data abstraction format was used to gather the required information from patients' records. A team of six trained midwives conducted the data collection, while two public health officers served as supervisors during the process.

Data were collected using a pretested, self-structured, interviewer-administered questionnaire and medical record review. The questionnaire was adapted from various relevant literature with necessary modifications tailored to the specific context of the study (2, 16, 19, 24, 27, 30, 32). A data abstraction format was used to gather the required information from patients’ records. A team of six trained midwives conducted the data collection, while two public health officers served as supervisors during the process. The questionnaire has been included as Supplementary Material for reference.

The questionnaire was first written in English, then translated into Afan Oromo, and finally back into English to check for consistency. To ensure the questionnaire and data collection methods matched the study's objectives, a pre-test was conducted with 5% of the total patient sample. Data collectors and supervisors were trained for two days on research ethics, data collection tools, and procedures to ensure high-quality data. During the data collection phase, ongoing supervision of data collectors occurred, and regular meetings were held involving data collectors, supervisors, and investigators. Participants were interviewed in private places to ensure their privacy and confidentiality as well as to encourage participation. Before data entry, collected data underwent review and checks to ensure completeness.

### 2.7 Data processing and statistical analysis

Following the coding and inputting of data into Epi Info version 7.2.6, the data were exported to the Statistical Package for Social Sciences (SPSS) version 26 for cleaning and analysis. Descriptive statistics including frequency, percentage, mean, and standard deviation were used to present key characteristics of the study population. The association between independent variables and dual contraceptive use was modeled using binary logistic regression analysis. In the bivariable logistic regression model, a significance level of 0.25 was set as a threshold to select variables for multivariable logistic regression analysis, aiming to control for confounding effects. The existence of multicollinearity among explanatory variables was assessed using the variance inflation factor (VIF). In this study, the mean VIF was 1.3, indicating that there was no multicollinearity. The model was fitted using the standard model-building approach. Hosmer and Lemeshow's goodness-of-fit test was used to assess the model's fitness. The diagnostic result indicated the model fit the data well (*p* = 0.86). In the multivariable logistic regression analysis, the adjusted odds ratio (AOR) with a 95% confidence interval (CI) was utilized to determine predictors of dual contraceptive use. At this level, variables with a *p*-value less than 0.05 were deemed statistically significant.

## 3 Result

### 3.1 Socio-demographic characteristics

Among the 347 sampled respondents, 342 completed the interview, yielding a response rate of 98.6%. The mean age of the participants was 33.52 (±5.16) years. One hundred five (30.7%) of the participants were in the age group of 30-34, and the majority, 212 (62% were urban residents. About 33.6% of women were able to read and write, and 156 (45.6%) were housewives (Table 1).

**Table 1:** Socio-demographic characteristics of women living with HIV receiving anti-retroviral therapy at public health facilities in boset district, Ethiopia, 2023 (*n* = 342).

Variables	Categories	Frequency	Percentage
Age	15–24	27	7.9
	25–29	73	21.3
	30–34	105	30.7
	35–39	95	27.8
	40–49	42	12.3
Place of residence	Urban	212	62.0
	Rural	130	38.0
Religion	Orthodox	152	44.4
	Protestant	99	28.9
	Muslim	73	21.4
	Others*	18	5.3
Marital status	Married	241	70.5
	Single	44	12.9
	Divorced	32	9.3
	Separated	25	7.3
Ethnicity	Oromo	262	76.6
	Amhara	49	14.3
	Gurage	20	5.9
	Others**	11	3.2
Women Educational status	No formal education	70	20.5
	Read and write	115	33.6
	Primary	95	27.8
	Secondary	36	10.5
	College and above	26	7.6
Spouse educational status (*N* = 298)	No formal education	40	13.4
	Read and write	45	15.1
	Primary	138	46.4
	Secondary	46	15.4
	College and above	29	9.7
Women occupation	Housewife	156	45.6
	Daily laborer	79	23.1
	Merchant	59	17.3
	Government employee	23	6.7
	Others***	25	7.3
Spouse occupation (*n* = 298)	Farmer	171	57.4
	Daily laborer	53	17.8
	Merchant	51	17.1
	Government employee	15	5.0
	Others****	8	2.7
Average monthly income	≤1,000 Ethiopian birr	141	41.2
	>1,000 Ethiopian birr	201	58.8

* Catholic, Wakefata, **Tigre, Wolaita, Silte, ***Student, Housemaid, Waitress, Female sex workers, ****non-governmental employee, driver, unemployed/jobless.

### 3.2 Sexual and reproductive health characteristics

In this study, 159 respondents (46.5%) reported having 1–2 children, while approximately two-thirds of the participants (217, 63.5%) expressed a desire to have more children. Among the respondents, 216 individuals (63.2%) had used some form of contraception since testing positive for HIV. Additionally, 173 participants (51.0%) demonstrated good knowledge of dual contraceptive methods (Table 2).

**Table 2:** Sexual and reproductive health-related characteristics of women living with HIV receiving anti-retroviral therapy at public health facilities in boset district, Ethiopia, 2023 (*n* = 342).

Variables	Categories	Frequency	Percentage
Number of children	0	49	14.3
	1–2	159	46.5
	3–4	105	30.7
	≥5	29	8.5
Women's desire to have more children	No	125	36.5
	Yes	217	63.5
Partner desire to have more children (*N* = 298)	No	101	33.9
	Yes	197	66.1
Ever used any contraceptive method since being HIV-positive	No	126	36.8
	Yes	216	63.2
Pregnancy since HIV positive	No	204	59.6
	Yes	138	40.4
Knowledge about dual contraceptive	Poor	169	49.0
	Good	173	51.0

### 3.3 Clinical and HIV-related characteristics

Out of all participants, only 53 (15.5%) reported having a history of other STIs in the past year, and the majority, 299 (87.4%), had single sexual partners. About 221 (74.2%) disclosed their HIV status to their partner (Table 3).

**Table 3:** Clinical and HIV related characteristics among women living with HIV receiving anti-retroviral therapy at public health facilities in boset district, Ethiopia, 2023(*n* = 342).

Variables	Categories	Frequency	Percentage
STI history in the past 12 months	No	289	84.5
	Yes	53	15.5
Number of sexual partners	1	299	87.4
	≥2	43	12.6
Know the HIV status of the partner (*n* = 298)	No	117	39.3
	Yes	181	60.7
Partner's HIV status (*n* = 181)	Positive	107	59.1
	Negative	74	40.9
Disclosed HIV status to partner (*n* = 298)	No	77	25.8
	Yes	221	74.2
Reason for not disclosing (*n* = 77)	Fear of divorce	57	74.0
	Fear of violence by a partner	47	61.0
	Fear of stigma and discrimination	17	22.1
Perceived disclosure of HIV status to a sexual partner is important	No	82	24.0
	Yes	260	76.0
Duration on ART (years)	≤5	189	55.3
	>5	153	44.7
Current CD4 cell count	<250	79	23.1
	250–349	71	20.8
	≥350	192	56.1
Current viral load	Suppressed	323	94.5
	Low viremia	10	2.9
	high viral load	9	2.6

CD4, clusters of differentiation 4; HIV, human immunodeficiency virus; STI, sexually transmitted infections.

### 3.4 Dual contraceptive use

The utilization of dual contraceptives among women living with HIV who are receiving antiretroviral therapy at public health facilities in the Boset district was found to be 33.7% (95% CI: 28–40). Among those who used dual contraceptives, the majority used condoms plus implants (54.9%), followed by condoms plus injectables (20.7%).

### 3.5 Factors associated with dual contraceptive use

Place of residence, women's desire to have more children, knowledge about dual contraceptives, STI history in the past 12 months, number of sexual partners, perceived disclosure of HIV to a sexual partner, and current CD4 cell count were the variables that fulfilled the criteria *P* < 0.25 and transferred to multivariable analysis. After adjusting for confounding variables in the multivariable analysis, place of residence, knowledge about dual contraceptives, STI history in the past 12 months, and the number of sexual partners were found to be statistically significant factors associated with dual contraceptive utilization.

Accordingly, the odds of dual contraceptive use were 2.5 times greater among urban residents compared with rural residents (AOR = 2.5, 95% CI: 1.97–8.08). Compared to women with poor knowledge of dual contraceptives those with good knowledge of dual contraceptives had nearly 4-fold higher odds of dual contraceptive utilization (AOR = 3.8, 95% CI: 2.36–8.67). Women who had a history of STIs in the past 12 months had 2.6 times higher (AOR = 2.6, 95% CI: 1.25–4.72) odds of dual contraceptive use than their counterparts. Moreover, the odds of dual contraceptive utilization were about twice as high among women with two or more sexual partners compared to those with a single sexual partner (AOR = 1.9, 95% CI: 1.18–6.68) (Table 4).

**Table 4:** Factors associated with dual contraceptive utilization among women living with HIV receiving anti-retroviral therapy at public health facilities in boset district, Ethiopia, 2023.

Variables	Categories	Dual contraceptive use		COR (95% CI)	AOR (95% CI)
		Yes (%)	No (%)		
Place of residence	Rural	30 (19.5)	100 (48.4)	1	1
	Urban	95 (80.5)	117 (51.6)	2.7 (2.01–7.27)*	2.5 (1.97–8.08)**
Women's desire to have more children	No	72 (47.4)	53 (27.9)	1	1
	Yes	80 (52.6)	137 (72.1)	0.4 (0.33–0.93)*	0.5 (0.52–1.35)
Knowledge about dual contraceptive	Poor	49 (30.6)	120 (65.9)	1	1
	Good	111 (69.4)	62 (34.1)	4.4 (2.51–8.14)*	3.8 (2.36–8.67)**
STI history in the past 12 months	No	94 (75.2)	195 (89.9)	1	1
	Yes	31 (24.8)	22 (10.1)	2.9 (2.17–7.19)*	2.6 (1.25–4.72)**
Number of sexual partners	1	83 (81.4)	216 (90.0)	1	1
	≥2	19 (18.6)	24 (10.0)	2.1 (1.22,5.31)*	1.9 (1.18–6.68)**
Perceived disclosure of HIV status to a sexual partner is important	No	40 (22.3)	42 (25.8)	1	
	Yes	139 (77.7)	121 (74.2)	1.2 (1.01–4,81)*	0.99 (0.76- 3.21)
Current CD4 cell count	<250	34 (21.8)	45 (24.2)	1	1
	250–349	24 (15.4)	47 (25.3)	0.7 (0.24–0.93)*	0.4 (0.16–1.10)
	≥350	98 (62.8)	94 (50.5)	1.4 (0.48–1.83)	1.1 (0.33–1.69)

AOR, adjusted odds ratio; CI, confidence interval; CD4, Clusters of differentiation 4; COR, crude odds ratio; HIV, human immunodeficiency virus; STI, sexually transmitted infections.

* Significant at *p*-value <0.25 in unadjusted logistic regression analysis.

** Significant at *p* < 0.05 in adjusted logistic regression analysis, 1 = Reference.

## 4 Discussion

This study revealed that the magnitude of dual contraceptive use was 33.7% (95% CI: 28–40), knowledge about dual contraceptives, STI history in the past 12 months, and number of sexual partners, were factors significantly associated with dual contraceptive use.

The current study revealed that the magnitude of dual contraceptive use was 33.7% (95% CI: 28–40). This finding is comparable to results from studies conducted in Thailand (29.6%), India (30%) (33), South Africa (33%) (34), Bungoma City, Kenya (38.5%) (16), Wolaita Zone, Southern Ethiopia (28.6%) (27), Hossana Hospital, Ethiopia (28.3%) (35), Gondar City, Northwest Ethiopia (28.8%) (30), Gimbie Town, Ethiopia (30%) (25), and Fitche Hospital, Ethiopia (32%) (26). However, it was higher than studies conducted in Switzerland (10.7%) (11), Zambia (17.7%), South Africa (6.8%), Mekelle, Ethiopia (15.7%) (21), Addis Ababa, Ethiopia (14.7%), West Showa Zone, Ethiopia (21.4%) (24), Gebretsadik Shawo Hospital, SNNPR, South West Ethiopia (19.8%) (23), Finote-Selam Hospital, Northwest Ethiopia (21.8%) (36), and Gondar Hospital, Ethiopia (13.2%) (20). The higher dual contraceptive use in this study compared to previous findings may be due to enhanced integration of family planning with HIV services, improved healthcare access, and increased awareness of dual protection. Differences in study periods, sample characteristics, and regional variations in contraceptive uptake could also explain the discrepancy.

Moreover, the use of dual contraceptives in this study was lower than in studies done in Brazil (72%) (37), Mumbai India (69%) (38), Bahirdar town, Ethiopia (64.2%) (28), and Bishoftu town, Ethiopia (56.9%) (19). This discrepancy may be attributed to variations in the sociodemographic and cultural characteristics of the study participants. Furthermore, inconsistencies between studies might arise from differences in sample size, study period, and the settings in which the studies were conducted.

Urban women had higher odds of using dual contraceptives compared to their rural counterparts. This finding is supported by studies conducted in Finote-Selam Hospital, Northwest Ethiopia (36), Hossana, Southern Ethiopia (32), Gondar City, Northwest Ethiopia (30), and West Showa Zone, Ethiopia (24). This could be attributed to disparities in access to information and contraceptive services. Urban residents have greater exposure to information and technologies related to contraceptives, which may facilitate their effective use. Furthermore, urban women are thought to have easier access to contraceptive methods (39).

Women with good knowledge of dual contraceptive methods had higher odds of using dual contraception compared to those with poor knowledge. This is in line with a study undertaken in Bungoma City, Kenya (16). Increased awareness and understanding of these methods likely empower women to make informed choices about their contraceptive practices, thereby enhancing their likelihood of use (40).

This study also revealed that the odds of dual contraceptive use were greater among women with a history of STI. This finding aligns with a study conducted in Wolaita zone, Southern Ethiopia (27). This may be attributed to increased risk awareness among women with a history of STIs, which motivates them to adopt dual contraceptive methods for comprehensive protection against both STIs and unintended pregnancies (41, 42).

Moreover, women who had multiple sexual partners had increased odds of using dual contraceptives compared to their counterparts. This finding is consistent with a study done in Hossana, Southern Ethiopia (32). This can be justified by the heightened awareness and perceived risk of STIs and unintended pregnancies among women with multiple partners. The need for additional protection against both STIs and pregnancy likely motivates these women to adopt dual contraceptive methods to ensure comprehensive protection (43). Conversely, findings from Finote-Selam Hospital, Northwest Ethiopia, showed that women with multiple sexual partners had lower odds of dual contraceptive utilization (36).

### 4.1 Implications for practice and policy

The findings of this study have important public health implications. The moderate level of dual contraceptive use indicates the need for intensified efforts to promote dual protection among WLHIV. Health education programs should emphasize the benefits of dual contraceptive use, particularly in rural areas, where access remains limited. Integrating reproductive health services with ART programs can enhance access to contraceptive counseling and increase uptake. Moreover, healthcare providers should leverage STI treatment visits as opportunities for reinforcing dual protection messages.

## 5 Limitations of the study

Being a cross-sectional study prevented the establishment of causal relationships between variables. Additionally, because it was conducted in health facilities, the findings may not apply to HIV-positive women outside the study area or those who do not access healthcare. The study also lacked qualitative data and did not include perspectives from sexual partners, limiting its depth. Furthermore, recall bias may have affected the accuracy of participants' reports on past contraceptive use or STI history. Social desirability bias could also be a concern, as some participants might have provided responses, they deemed socially acceptable rather than truthful.

## 6 Conclusion

In this study, one-third of WLHIV on ART utilized dual contraceptives. Factors such as place of residence, knowledge about dual contraceptives, history of STIs in the past 12 months, and number of sexual partners were identified as statistically significant factors associated with dual contraceptive use. The findings suggest the need for targeted educational programs to enhance awareness and knowledge about dual contraceptives, especially in rural areas, and expanding access to contraceptive services in rural and underserved areas are crucial steps forward.

## Data Availability

The raw data supporting the conclusions of this article will be made available by the authors, without undue reservation.
