# Supplementary material for: Dual contraceptive use among women living with HIV on anti-retroviral therapy in Boset district, Ethiopia
Source: Front Glob Womens Health. 2025 Apr 1;6:1510299. doi: 10.3389/fgwh.2025.1510299 (PMC11996836; doi:10.3389/fgwh.2025.1510299)
Supplement: Supplementary file 1 [file Table1.docx]

QUESTIONNAIRES-ENGLISH VERSION

Questionnaire (English Version Questionnaire)

Questionnaire: **Dual Contraceptive Use among** **Women Living with HIV on Anti-Retroviral Therapy in Boset District, Ethiopia**

Code No._____________________________________

Interviewer's name____________________________________

Supervisor's name____________________________________

Name of health facility --------------------------------------------------

Date of interview dd---------mm--------- yy -----------

Instruction: - Encircle the responses for questions with alternatives and for open ended questions write on the space provided.

| **Part I: Socio-demographic and economic characteristics** | | | | | |
| --- | --- | --- | --- | --- | --- |
| **S.NO.** | **Questions** | | **Response** | | **Skip to /Remark** |
| 101 | How old are you? | | 1.______ years | |  |
| 102 | Where is your residence? | | 1. Urban  2. Rural | |  |
| 103 | What is your religion? | | 1. Orthodox  2. Muslim  3. Catholics  4. Protestant  5. Other (specify)……… | |  |
| 104 | What is your marital status? | | 1. Married  2. Single  3. Divorced  4.Widowed  5. Separated | |  |
| 105 | What is your ethnicity? | | 1. Amhara  2. Oromo  3. Tigray  4. Others (specify)……… | |  |
| 106 | What is your educational status? | | 1. Unable to read and write  2. Can read and write only  3. Primary education  4. Secondary education  5. College diploma and above | |  |
| 107 | What is your husband educational status? | | 1. Unable to read and write  2. Can read and write only  3. Primary education  4. Secondary education  5. College diploma and above | |  |
| 108 | What is your occupational status? | | 1. Student  2. Merchant  3. Government employ  4. Housewife  5. Daily labourer  6. Others (specify)……… | |  |
| 109 | What is your husband occupational status? | | 1.Student  2. Merchant  3. Government employ  4.. Daily labourer  5. Others (specify)…….. | |  |
| 110 | Family monthly income? | | 1. _______ETB(Birr) | |  |
| **Part II : Sexual and reproductive factors** | | | | | |
| S.No | Questions | | Answer/Code | |  |
| 200 | Now I would like to talk about family planning method - the various ways or methods that a couple can use to delay or avoid a pregnancy. Have you ever heard of it/them (FAMILY PLANNING METHODS)? | |  | |  |
| 201 | Female Sterilization.  PROBE: Women can have an operation to avoid having any more children. | | 1.Yes  2. No | |  |
| 202 | Male Sterilization.  PROBE: Men can have an operation to avoid having any more children. | | 1.Yes  2. No | |  |
| 203 | Intrauterine contraceptive devices (IUCDs).  PROBE: Women can have a loop or coil placed inside them by a doctor or a nurse which can prevent pregnancy for one or more years. | | 1.Yes  2. No | |  |
| 204 | Injectable.  PROBE: Women can have an injection by a health provider that stops them from becoming pregnant for one or more months. | | 1.Yes  2. No | |  |
| 205 | Implants.  PROBE: Women can have one or more small rods placed in their upper arm by a doctor or nurse which can prevent pregnancy for one or more years. | | 1.Yes  2. No | |  |
| 206 | Pills.  PROBE: Women can take a pill every day to avoid becoming pregnant | | 1.Yes  2. No | |  |
| 207 | Male Condom.  PROBE: Men can put a rubber sheath on their penis before sexual intercourse. | | 1.Yes  2. No | |  |
| 208 | Female Condom.  PROBE: Women can place a sheath in their vagina before sexual intercourse. | | 1.Yes  2. No | |  |
| 209 | Emergency Contraception.  PROBE: As an emergency measure, within three days after they have unprotected sexual intercourse, women can take special pills to prevent pregnancy. | | 1.Yes  2. No | |  |
| 210 | Standard Days Method.  PROBE: A woman uses a string of colored beads to know the days she can get pregnant. On the days she can get pregnant; she uses a condom or does not have sexual intercourse. | | 1.Yes  2. No | |  |
| 211 | Lactational Amenorrhea Method (LAM).  PROBE: Up to six months after childbirth, before the menstrual period has returned, women use a method requiring frequent exclusive breastfeeding day and night. | | 1.Yes  2. No | |  |
| 212 | Withdrawal.  PROBE: Men can be careful and pull out before reaching at climax. | | 1.Yes  2. No | |  |
| 213 | Dual contraceptive method.  Use of any hormonal, IUCD or permanent modern contraceptive method along with male or female condom | | 1. Yes  2. No | |  |
|  | **Knowledge question** | |  | |  |
| 214 | Have you heard of any other ways or methods that women or men can use to avoid pregnancy? | | 1. Yes (specify)…………  2. No | |  |
| 215 | If yes for question # 2013, Do you think HIV positive women should use condom with other modern contraceptive method? | | 1. Yes 2. No | |  |
| 216 | Do you think dual contraceptive method prevent unintended pregnancy? | | 1. Yes 2. No | |  |
| 217 | Do you think dual contraceptive method prevent HIV transmission to sexual partner/s? | | 1. Yes 2. No | |  |
| 216 | Do you think dual contraceptive method prevent new strain of virus? | | 1. Yes 2. No | |  |
| 217 | Do you think dual contraceptive method prevent other STI transmission? | | 1. Yes 2. No | |  |
| 218 | Do you think dual contraceptive method prevent high viral load result? | | 1. Yes 2. No | |  |
| 219 | If yes for question # 218, Have you ever used any contraceptive method since you aware your HIV status? | | 1. Yes 2. No | |  |
| 220 | If yes for question # 219, Have you ever used condom since you aware your HIV status? | | 1. Yes 2. No | |  |
| 221 | If yes for question # 219, Have you ever used any other modern contraceptive method with condom since you aware your HIV status?? | | 1. Yes 2. No | |  |
| 222 | If yes for question # 221, are you currently using any contraceptive method? | | 1. Yes 2. No | |  |
| 223 | If yes for question # 222, are you currently using condom? | | 1. Yes 2. No | |  |
| 224 | If yes for question # 223, are you currently using any other modern contraceptive method with condom? | | 1. Yes 2. No | |  |
| 225 | If yes for question # 224, what main contraceptive method do you use with condom? | | 1. Pill 2. Injectable 3. Implant 4. IUCD 5. Permanent 6. Others (specify)…… | |  |
| 226 | If yes for question # 225, did you use a condom every time you had sexual intercourse? | | 1. Yes 2. No | |  |
| 227 | If you are using dual contraceptive method currently, why do you choose? | | 1. To prevent unintended pregnancy 2. To prevent HIV to sexual partner 3. To prevent new strain of virus 4. To prevent other STI 5. To avoid high viral load 6. Others (specify)… | |  |
| 228 | If you are not using dual contraceptive method currently, why? | | 1. Side effect of contraceptive 2. Wanting to have children 3. Not disclosing HIV status to sexual partner 4. Same HIV status with partner 5. Unwillingness sexual partner 6. Not accessibility of the method 7. Other (specify)…………….. | |  |
| 229 | How many live children do you have? | | 1. Write in number----------- | |  |
| 230 | Do you have desire to have more child in the future? | | 1. Yes 2. No | |  |
| 231 | Do your partner have desire to have more child in the future? | | 1. Yes 2. No | |  |
| **Part III: Clinical and HIV related factors** | | | | | |
| S.No | Questions | Answer/Code | |  | |
| 301 | Did you have other STI history or symptoms of STI (a bad-smelling, abnormal discharge from the Vagina or a genital sore or ulcer)? | 1. Yes 2. No 3. I can’t remember/I don’t know | |  | |
| 302 | If yes for question # 301, have you been treated? | 1. Yes 2. No | |  | |
| 303 | If no for question # 302, why do you not treated? | 1. No access of treatment 2. No need of treatment 3. No money for payment 4. Other (specify)………. | |  | |
| 304 | How many sexual partners do you have? | 1. Write in number----------- | |  | |
| 305 | Do you know HIV status of your regular partner? | 1. Yes 2. No | |  | |
| 306 | If yes for question # 305, what is HIV status of your sexual partner? | 1. Positive 2. Negative | |  | |
| 307 | Do you disclose your HIV status to your sexual partner? | 1. Yes 2. No | |  | |
| 308 | If no for question # 307, what was/were the reason/s?  (More than one answer is possible) | 1. Fear of divorce 2. Fear of violence by partner 3. Fear of stigma and discrimination 4. Other (specify)………. | |  | |
| 309 | Do you think disclosure of HIV status to sexual partner is important? | 1. Yes 2. No | |  | |
| 310 | If yes for question # 309, What is the advantage of HIV status disclosure to sexual partner?  (More than one answer is possible) | 1. To support each other 2. To protect sexual partner from HIV 3. To attend better care 4. To practice safe sex 5. To avoid unintended pregnancy 6. To discuss freely 7. Other (specify)………. | |  | |

**Thank you so much for your time**

| Part IV: Chart review | | |
| --- | --- | --- |
| 401 | Duration of Antiretroviral therapy (ART) | 1. In numbers-------Years |
| 402 | Current CD4 cell count | 1. In numbers---------- |
| 403 | Current viral load result | 1. In numbers---------- |
| 404 | Other STI history (since the client is aware of her HIV status) | 1. Yes 2. No |
